# Chronic Posttraumatic Epilepsy following Neocortical Undercut Lesion in Mice

**DOI:** 10.1371/journal.pone.0158231

**Published:** 2016-06-27

**Authors:** Xingjie Ping, Xiaoming Jin

**Affiliations:** Department of Anatomy and Cell Biology, Stark Neurosciences Research Institute, Indiana Spinal Cord and Brain Injury Research Group, Indiana University School of Medicine, 320 W. 15^th^ Street, Indianapolis, Indiana, 46202, United States of America; University of California, Riverside, UNITED STATES

## Abstract

Posttraumatic epilepsy (PTE) usually develops in a small percentage of patients of traumatic brain injury after a varying latent period. Modeling this chronic neurological condition in rodents is time consuming and inefficient, which constitutes a significant obstacle in studying its mechanism and discovering novel therapeutics for its prevention and treatment. Partially isolated neocortex, or undercut, is known to induce cortical hyperexcitability and epileptiform activity *in vitro*, and has been used extensively for studying the neurophysiological mechanism of posttraumatic epileptogenesis. However, whether the undercut lesion in rodents causes chronic epileptic seizures has not been systematically characterized. Here we used a miniature telemetry system to continuously monitor electroencephalography (EEG) in adult C57BL mice for up to 3 months after undercut surgery. We found that 50% of animals developed spontaneous seizures between 16–50 days after injury. The mean seizure duration was 8.9±3.6 seconds, and the average seizure frequency was 0.17±0.17 times per day. There was no progression in seizure frequency and duration over the recording period. Video monitoring revealed behavioral arrests and clonic limb movement during seizure attacks. A pentylenetetrazol (PTZ) test further showed increased seizure susceptibility in the undercut mice. We conclude that undercut lesion in mice is a model of chronic PTE that involves spontaneous epileptic seizures.

## Introduction

Of the 1.7 million victims of traumatic brain injury (TBI) in the United States annually, 5–53% of them, depending on the type and severity of brain injury, will develop chronic epileptic seizures [1, 2]. Posttraumatic epilepsy (PTE) accounts for 20% of symptomatic epilepsy in the general population. It is poorly controlled by currently available antiepileptic drugs[1, 3], and constitutes one of the major conditions that compromise functional outcome and quality-of-life in TBI patients[4, 5][6]. PTE has been observed and characterized in several animal models of TBI, such as lateral fluid percussion, controlled cortical impact, and weight drop injury[7–11]. These models are useful for studying the mechanisms of posttraumatic epileptogenesis [12]. However, the low incidences of spontaneous seizures and the requirement for long periods of video/EEG monitoring in these models makes it very inefficient to use them for evaluating therapeutic or prophylactic efficacy. Developing and validating efficient models of PTE are important and urgent for research on PTE.

Partially isolated neocortex, or undercut, is a well-established animal model of posttraumatic epileptogenesis [13, 14]. It is created by surgically making a transcortical cut and an undercut in the white matter so that a portion of the neocortex is injured and deprived of afferent input, and eventually becomes hyperexcitable. Presumably, the lesion models penetrating brain injury in humans, in which up to 53% of penetrating TBI patients developed epilepsy [2, 12, 15]. In cats and rodents, epileptiform discharges become apparent after a latency of 2–3 weeks, and are detectable with field potential recording in brain slices in up to 93% of undercut rats [13, 16–18]. In humans, spontaneous seizures developed in 29% of patients who received frontal lobe undercut surgery for controlling intractable psychiatric conditions [19]. Extensive *in vivo* or *in vitro* work on the neurophysiology of PTE has been done in this model in cats [14, 17, 20] and in rodents [21–26]. For example, the roles of axon sprouting and excitatory hyper-connectivity, homeostatic activity regulation, impaired chloride homeostasis and GABAergic inhibition in PTE, and the prevention of PTE through releasing tetrodotoxin have been demonstrated in or proposed based on studies in this model [17, 21, 27, 28]. Furthermore, the undercut model is highly efficient because epileptiform activity can be evoked in almost all rats 2 weeks after injury [13]. In cortical slices prepared from undercut rats, spontaneous and evoked epileptiform discharges can be recorded in the majority of slices after 2 weeks following injury [13]. However, although anecdotal observations of electrographic and behavioral seizures were made in a few undercut rats, systematic characterization of chronic epileptic seizure of this model in rodents has not been completed [21, 29]. The lack of this critical piece of information substantially undermines the validity of the model and limits its use in epilepsy research.

Here we used a wireless electroencephalogram (EEG) recording system to continuously monitor spontaneous epileptiform activities in undercut mice for 1.5 to 3 months. We found that half of the undercut mice developed spontaneous seizures and epileptiform spikes, which were accompanied by behavioral changes and reduction in the threshold of seizure induction. Our data support that undercut is a model of chronic PTE.

## Materials and Methods

### Animals

Forty four male C57/BL6 mice at the age of 2 months were used in this experiment: fourteen for EEG recording and thirty for pentylenetetrazol (PTZ) test. The mice were housed 5 per cage in a temperature- and humidity-controlled animal facility on a 12-hour light/dark cycle, with food and water supplied *ad libitum*. All procedures were approved by the Animal Care and Use Committee of the Institutional Guide for the Care and Use of Laboratory Animals at Indiana University School of Medicine.

### Undercut surgery

An undercut lesion was made as previously described [30, 31]. An undercut device was made consisting of a supporting plate, a guiding tube, and a syringe needle that was bent 90° at 2–3 mm from the tip. Animals were anesthetized with ketamine and xylazine (87.7/12.3 mg/kg, *i*. *p*.) and fixed on a stereotaxic apparatus. Following exposing the skull with a midline incision, a rectangular groove (2×4 mm) was drilled on the center of the left skull above the left sensorimotor cortex and the central piece of bone was removed. The needle of the undercut device was placed in the middle of the cranial window in a parasagittal direction, 1 mm lateral to the sagittal suture and inserted in a horizontal direction through the dura and slowly lowered to 1.2 mm depth to create a transcortical cut. It was then rotated 135° away from the midline to create a half-circle white matter/deep layer VI undercut. The needle was raised again to underneath the pia and withdrawn. A piece of sterile plastic film was placed on the cranial window to cover the exposed cortex, and the scalp was sutured.

### Transmitter implantation

We used a telemeter system for continuous EEG recording in mice. The system consisted of a miniature transmitter (~1 gram weight) and a capacitive-coupled receiver (Epoch system, Epitel, Utah)[32]. The transmitter is composed of a physiological amplifier controlling a frequency modulation oscillator encased by optically clear epoxy. The receiver amplifies and filters EEG signals via a frequency-to-voltage converter and a band-pass filter in it. The system had a bandwidth of 0.1–120 Hz and a gain of 4000x. The two leads of the transmitter provided single channel EEG signals in a differential mode.

The transmitters were implanted 2 weeks after undercut surgery, a time point at which cortical hyperexcitability is apparent after a latent period of posttraumatic epileptogenesis in this model [9]. Animals were anesthetized with ketamine and xylazine (87.7/12.3 mg/kg, *i*.*p*.) and fixed on a stereotaxic apparatus. After a midline incision of the scalp, the skin was pulled aside and the periosteum was removed with sterile cotton swabs. A small hole was drilled on the skull of the hemisphere contralateral to the undercut, ~1 mm lateral to the midline and parallel to the center of the craniotomy window of the undercut lesion. After the two wires of the transmitter were cut ~1–1.5 mm to length, one wire was inserted into the burr hole of the contralateral hemisphere, and the other passed through a hole at the center of a plastic film that covered the undercut cortex. The tips of the wires extended through the burr holes in the skull and into the cortex no more than 500 μm. The transmitter was then attached onto the skull by applying cyanoacrylate glue to its bottom and edge. The scalp was then sutured, and the animals were injected with 0.1 ml saline (i. p.) and allowed to recover on a heat pad until becoming awake. For uninjured control mice, transmitters were implanted on a similar location as undercut mice.

### Continuous video-EEG recording and histological verification of recording sites

The EEG signal from the receivers were converted into digital signals (Digidata 1440A, Axon Instruments, CA) and recorded to a computer. The mice were continuously monitored for 50 days (group I) or 90 days (group II). Sampling rate was set at 250 Hz. A digital video camera was used to monitor the behavior of the mice in their home cage.

At the end of video-EEG recording, animals were perfused with 0.9% NaCl followed by 4% Paraformaldehyde. The brains were removed, and cortical region containing the recording site was sectioned at a thickness of 30 μm using a cryostat (Leica CM1950, Leica Biosystems, IL). The sections were stained with Cresyl violet staining and imaged under a microscope to confirm the lesion location and recording sites.

### Pentylenetetrazol test

PTZ test was performed in two groups of undercut and naïve mice (n = 15 for each group) in 15 days after undercut surgery or at the same age using a published protocol [33]. These mice did not receive chronic EEG monitoring. A mouse was placed in a transparent box for 15 minutes to calm down, then an initial dose of PTZ (20 mg/kg, *i*.*p*. Sigma-Aldrich, St. Louis, MO, USA) was injected and the mouse was observed for 15 minutes. Thereafter, additional doses of 10 mg/kg of PTZ were given every 15 minutes until a convulsive seizure was observed. The total time to convulsive seizure and cumulative dose of PTZ injected were recorded.

### Data analysis

The EEG traces were manually analyzed. Similar to previously reported results, we observed transient loss of signals from time to time [26]. However, these short periods of signal loss can be easily identified and were excluded from data analysis. Seizures were identified as repetitive spike discharges with high amplitude (at least twice of baseline), and longer than 5 seconds duration [8]. Interictal spikes were identified as high amplitude sharp epileptiform waveforms with an interval between spikes at 1–8 seconds [34]. Video recordings were viewed to identify seizures and to determine behavioral changes during the time periods of electrographic seizures. The latency to first seizure, frequency of seizure events, and seizure duration were calculated based on data from the first seizure to the end of the recording period.

## Results

### Characteristics of undercut induced spontaneous seizures

**Fig 1:**
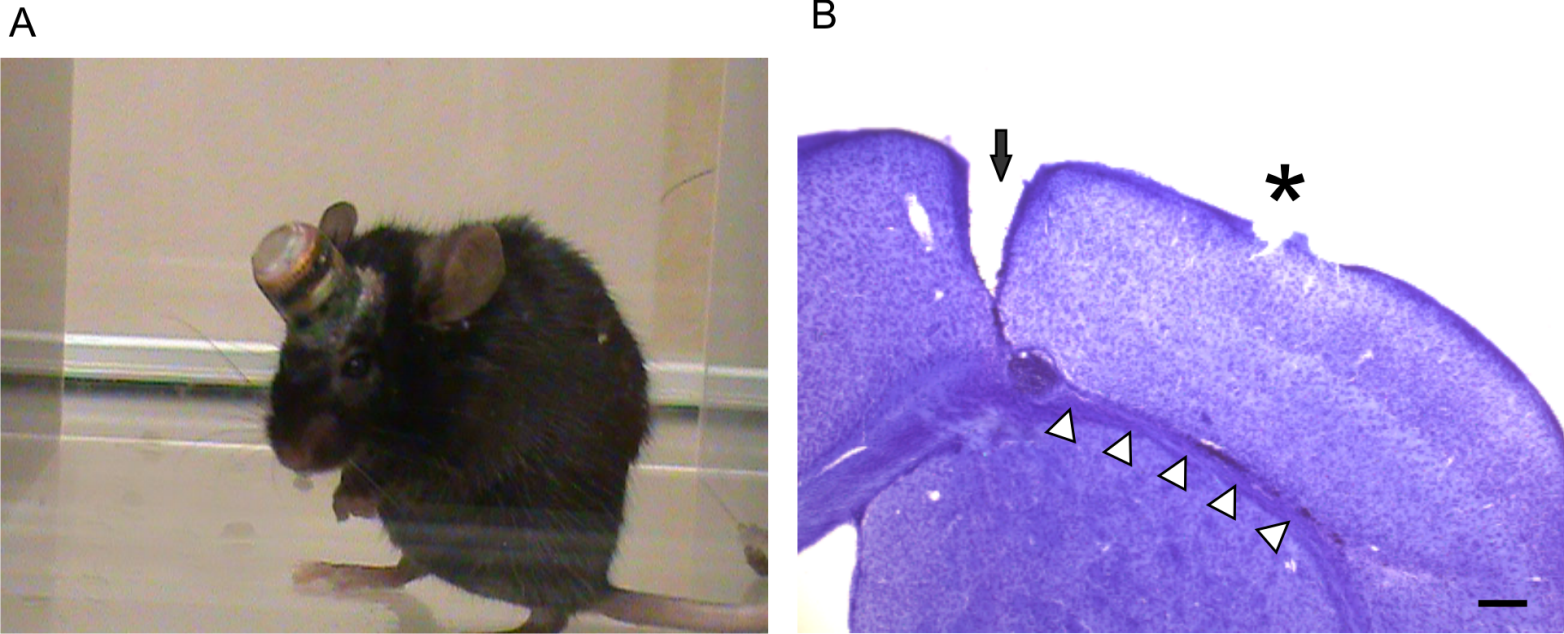
Chronic electroencephalography (EEG) monitoring with a telemetry system in undercut mice. (A). A mouse that was chronically implanted with a miniature transmitter showed normal behaviors during the monitoring period. (B). Histological verification of undercut lesion and recording site with Cresyl violet staining. A coronal brain section shows a continuous transcortical cut (black arrow) and an undercut (white triangles) lesion at 8 weeks after undercut surgery and the recording site located on the pial surface above the undercut lesion (asterisk). Scale bar in (B): 200 μm.

A total of 6 naïve adult C57BL mice and 14 undercut mice received continuous video-EEG monitoring for 50–90 days after injury. Implantation of the transmitters did not significantly interfere with general activity and behavior of the mice (Fig 1A) [35]. Nissl staining of brain tissue at the end of EEG recording confirmed that the undercut lesions in all mice were neat and within layer VI and whiter matter border. In some mice, the healing of the lesion was so complete that the transcortical and undercut sites were barely discernible. All the recording sites of the transmitter electrodes were located above the cortical lesion area (Fig 1B). No animal died during the surgeries or recording period.

**Fig 2:**
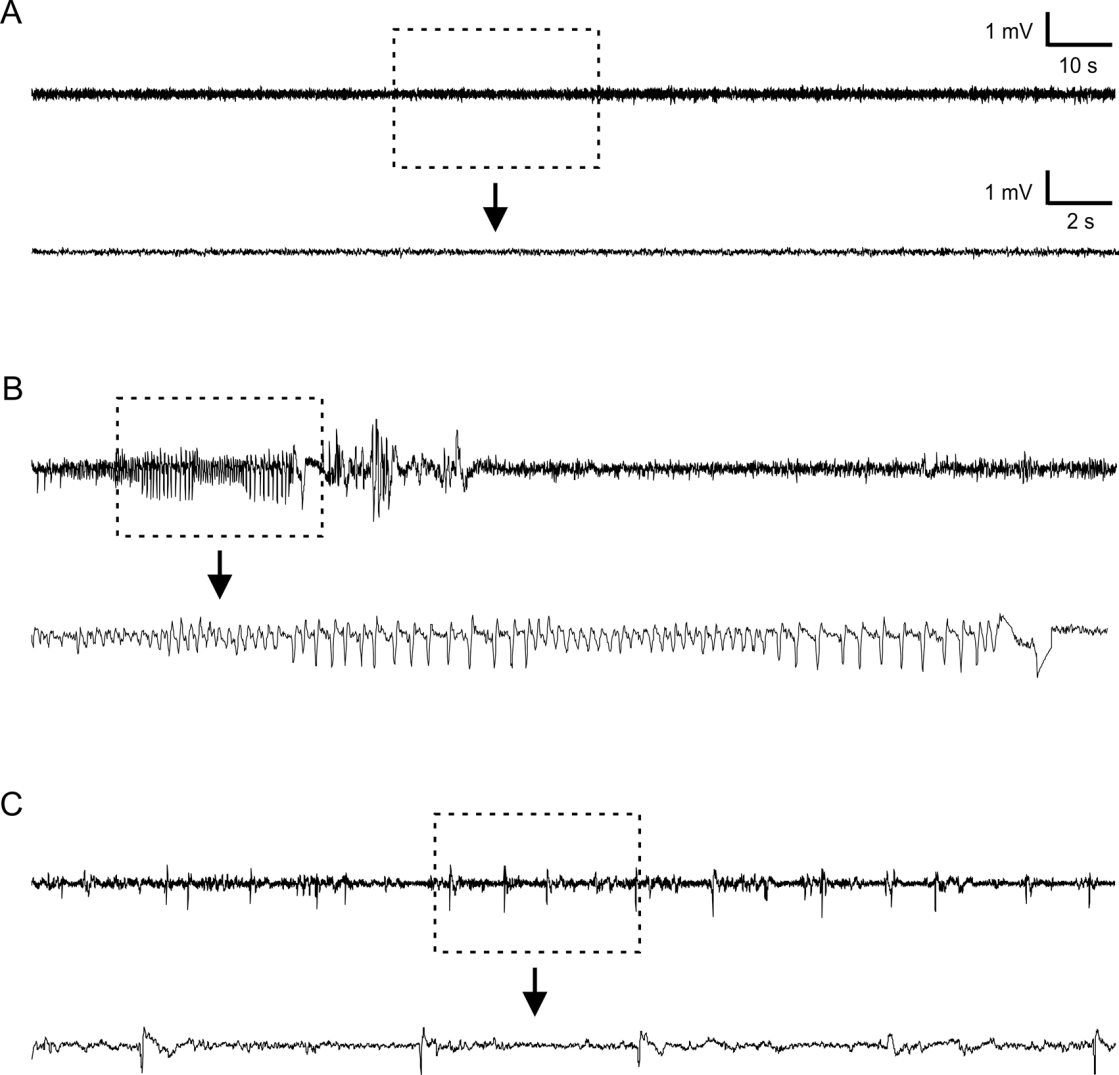
Representative EEG traces showing normal activity in a naïve mouse, and spontaneous seizure and interictal spikes in undercut mice. (A). A trace recorded in a naïve mouse. (B). A representative EEG trace of a spontaneous seizure that lasted for ~13 seconds in an undercut mouse at 51 days after injury. (C). A trace showing interictal spikes in an undercut mouse. Each bottom trace in A-C shows the rectangular area in an expanded time scale.

The mice were monitored in 2 groups with different recording periods. In the first group that was recorded for 50 days, 33.3% of the mice (2 of 6) developed spontaneous seizures. In the second group that was recorded for 90 days, 62.5% of them (5 of 8) developed spontaneous seizures. The total average of epileptic mice was 50% (7 of 14) (Table 1). All 7 mice that developed spontaneous seizure displayed a stereotypical EEG pattern including a cluster of high amplitude (at least twice of baseline), repetitive bursts of spikes (Fig 2B), with average duration of 8.9±3.6 s (Table 1). A typical electrographic seizure started with increasingly short interval interictal spikes, which turned into high amplitude rhythmic ictal activity dominated with theta activity (in the range of 4–8 Hz). The ictal activities usually lasted for 6-18s. The seizure events usually ended with a long-duration negative peak followed by irregular spikes with gradually decreasing amplitude (Fig 2B). Epileptic spikes were seen in 66.7% (4 of 6) and 75% (6 of 8) of mice of the first and second groups respectively, which led to the incidence of epileptiform spiking of 71.4% in all animals (Table 1).

**Table 1:** Characterization of development of epileptiform activity after undercut

Group	Recording time (days)	Epileptic mice (%)	Latency (days)	Frequency (seizure/day)	Seizure duration (s)	Spike (%)
1 (n = 6)	50	2 (33.3%)	20.5±0.7	0.13±0.26	8.2±4.3	4 (66.7%)
2 (n = 8)	90	5 (62.5%)	24.2±8.5	0.20±0.20	9.0±3.9	6 (75%)
Total (n = 14)		7 (50.0%)	23.1±7.2	0.17±0.17	8.9±3.6	10 (71.4%)

Most mice that had spontaneous electrographic seizures also had behavioral seizures, which were characterized by sudden behavioral arrest, head nodding, and clonic movements of forelimbs. All the seven animals that had spontaneous seizure also showed interictal spikes (Fig 2C). However, no behavioral abnormalities were observed during the occurrence of interictal spikes.

### Seizure development and progression

**Fig 3:**
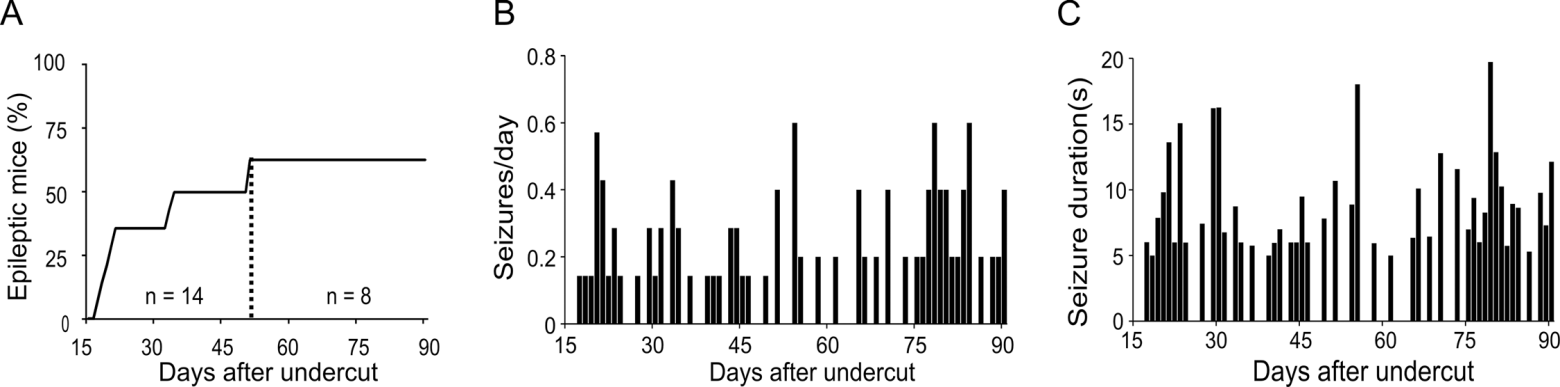
Characterization of spontaneous epileptic seizures in the undercut model. (A). Cumulative percentage of mice that developed posttraumatic epilepsy after undercut. In group I, 6 mice were recorded for 50 days; In group II, 8 mice were recorded for 90 days after injury. (B). Mean daily frequency of spontaneous seizure events per mouse during the recording period. There seems no significant change in the distribution of seizure frequency over time. (C). Mean duration of seizure events during the recording period. The seizure duration ranged between ~5–20 seconds.

In the 7 animals that developed spontaneous seizures, the latency to first seizure was 23.1 ± 7.2 days after undercut lesion (Table 1, 20.5 ± 0.7 days for group I and 24.2 ± 8.5 days for group II), ranging between 17–34 days. Of the epileptic mice, 5 of 7 started to show seizure activity within 3 weeks after undercut, and the other 2 mice showed first seizure events around 1 month post-injury (Fig 3A). The frequency of spontaneous seizures was quite low, with an average of 0.17 ± 0.17/day/mouse (Table 1). All mice had seizure free days (Fig 3B). The distribution of seizure frequency displayed a random pattern during the whole monitoring period and there was no progression in the frequency of seizures over time. The duration of seizure events was 8.9 ± 3.6 s (Table 1) and remained relatively consistent during the whole recording period (Fig 3C).

### Increase seizure susceptibility in PTZ test

**Fig 4:**
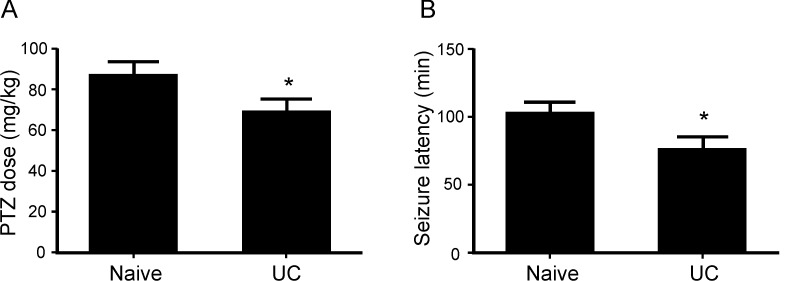
Reduced seizure threshold in undercut mice. Undercut mice received PTZ test at 15 days after undercut lesion. There were significant decreases in the cumulative dose of PTZ for seizure induction (A) and the latency period to first seizure (B) in the undercut than in the naïve mice. *: p<0.05, Student *t*-test.

PTZ tests were made in 15 naïve and 15 undercut mice. There was a significant increase in seizure susceptibility in the undercut mice. The cumulative PTZ dose needed for inducing convulsive seizure was significantly lower in undercut mice than in naïve mice (87.3±6.2 mg/kg and 69.3±6.1 mg/kg for naïve and undercut mice respectively, *p*<0.05, Student *t*-test), while the latency from initial dose of PTZ to the occurrence of convulsive seizure was significantly shorter in the undercut mice than naïve mice (103.3±8.2 minutes and 76.7±8.6 minutes for the naïve and undercut mice respectively, *p*<0.05) (Fig 4).

## Discussion

The present study used continuous video/EEG monitoring to characterize the development of spontaneous epileptic seizures and change in seizure susceptibility in the undercut model in mice. The major finding is that posttraumatic epilepsy developed in up to 62.5% of the undercut mice in 3–5 weeks after initial lesion. The spontaneous electrographical seizures were accompanied by freeze and clonic limb movement. A PTZ test also showed a significant decrease in the threshold of seizure induction. These results suggest that undercut lesion is a valid and efficient model of posttraumatic epilepsy that involves spontaneous seizures in about a half of the injured mice.

### Recording spontaneous epileptic seizures in undercut mice

The miniature telemetry system used in this study has been introduced and used for chronically recording seizure activities in neonatal and adult rodents [35]. This system is minimally invasive during surgical implantation, allows free moving of the animals so that long-term continuous recording is possible, and provides high quality recording without significant environmental interference. Therefore, it provides unique advantages for characterizing the development of posttraumatic epilepsy in rodent models. One limitation is that this system has only one channel, which does not allow determining the origin and propagation of epileptic events in cortical hemispheres. However, this limitation has been improved with the recent development of a new two-channel system by the company.

The spontaneous seizures in undercut mice featured high-amplitude rhythmic discharges that lasted for > 5s (Fig 2B)[8], which were accompanied by motor manifestation including behavioral arrest and subtle limb clonus. Development of posttraumatic epilepsy has been characterized in several models of TBI such as controlled cortical impact (CCI) and fluid percussion injury in rats and mice [8, 12, 34]. In the CCI and FPI models, the incidence of spontaneous seizures in rats varies from 3% to 50%, depending on the type, location, and severity of lesion; and the latencies from TBI to initial seizure are often several months [8, 9, 12]. In this study, the percentages of epileptic mice are 33.3% and 62.5% in the 2 groups of mice respectively, with a total incidence of 50%. Although the latent period we observed was mostly within 3–4 weeks (Fig 3A) after undercut lesion, the possibility of seizure events in an earlier time period cannot be excluded. In fact, acute seizures are observed within the first few days to one week after undercut injury or in other models of TBI [36, 37]. Because acute and chronic posttraumatic seizures are believed to be different in mechanism and clinical treatment [37, 38], we did not attempt to characterize early seizures within 2 weeks after undercut. The frequency of spontaneous seizures in undercut mice is 0.17 seizure per day (Fig 3B), which is comparable to the frequencies of up to 0.3 seizure per day in CCI and FPI models [12]. Seizure durations in the CCI and FPI models can last from dozens of seconds to hundreds of seconds [12, 39], which are much longer than what has been seen in the undercut mice (8.9±3.6 s) (Fig 3C).

A major factor that may contribute to a difference in seizure duration is injury type and severity. Specifically, severe TBI in FPI and CCI models leads to a more severe brain trauma and larger lesion area, and subsequently more dramatic and widespread pathological reaction and circuit reorganization, which may have a significant contribution to the long seizure duration [7]. Indeed, seizure duration of posttraumatic epilepsy has been shown to vary between a few seconds in fluid percussion and undercut to tens of seconds or over a hundred seconds in CCI and severe lateral fluid percussion models in rodents [40–42]. In addition, epileptiform spikes are detected in a large percentage of undercut mice, and reduced PTZ dosage is needed to induce general seizure in a shorter latency period. These findings are consistent with observations in other models of PTE and indicative of cortical hyperexcitability and decreased seizure threshold *in vivo* [43]. Recently, spontaneous electrographic and behavioral seizures have also been demonstrated in a high percentage of undercut mice at old age by another group [29], which further confirm our observation in this study and support the use of the rodent model for epilepsy research.

### Neocortical undercut as a model of PTE

Neocortical undercut lesion has been used as a model of posttraumatic epileptogenesis for studying the mechanisms of epileptogenesis *in vitro* and *in vivo* for decades [13, 23, 30]. In human, surgical undercut lesion to the white matter of the frontal cortex was used as a therapeutic approach for psychiatric diseases and was found to result in spontaneous seizures in about 40% of patients [19]. Undercut lesion of the parietal cortex in cats causes spontaneous seizures, which are accompanied by reduction in interneuron density, homeostatic synaptic plasticity, and development of synchronized network activity [17, 44]. In rats, evoked and spontaneous epileptiform discharges were recorded in undercut cortical slices of in 2 weeks after injury [13]. The development of a rodent model greatly enhances its application for basic study on the neurophysiological mechanism of posttraumatic epileptogenesis.

The neocortical undercut model is often used for studying the mechanisms of hyperexcitability and epileptogenesis. Histological studies showed a disorganized cortical cytoarchitecture which lacks normal arrangement of neurons on layers and columns and degeneration of layer V pyramidal neurons, as well as increased axonal length and number of axonal collaterals after undercut injury [26, 30, 44]. While electrophysiological experiments found that layer V pyramidal neurons received increased AMPA receptor-mediated excitatory synaptic drive and decreased GABA_A_ receptor-mediated inhibition, impaired chloride homeostasis, and formation of recurrent excitatory circuits in this undercut cortical slices [25]. These changes are believed to shift the balance within cortical circuits toward increased synaptic excitation and contribute to epileptogenesis [23].

Although spontaneous seizures have been reported in humans, monkeys, cats, and occasionally in rats [10, 31], they have not been systematically documented in rodent preparation. Results from the current study in mice indicate that undercut lesion dose increase seizure susceptibility and cause chronic posttraumatic epilepsy in a relatively short latent period. These findings fill the information gap about this well-studied model and support the use of this preparation as a valid rodent model of PTE.

### Advantages and limitations of the undercut model

The advantages of the undercut model include its easy preparation of the model, a high rate of epileptiform activity *in vitro* (epileptiform activity can be evoked in up to ~95% of cortical slices), high reproducibility, and a low rate of animal mortalities after injury. Now we further show that a good percentage of undercut mice develop spontaneous seizures *in vivo*.

In contrast to other PTE models that mimic the physical impact in TBI, the undercut lesion is apparently “artificial” and rarely seen in TBI patients. The surgical focal undercut lesion may be regarded both as a limitation and an advantage of this model. On one hand, the lesion does not mimic common clinical TBI such as those caused by falls, motor vehicle-related collisions, sports injuries, and explosive blasts. In these injuries, biomechanical forces are various and often induce simultaneous injury to remote brain structures including the hippocampus [9, 10, 40, 45, 46]. Although the undercut lesion can be regarded as a model of TBI because it involves tissue penetration and tissue loss, bleeding, and neuronal damage, which are followed by edema, inflammation, and other pathological reactions, it likely does not reproduce all aspects of penetrating TBI in humans. For example, cortical tissue damage and loss are limited after undercut, and other brain structures such as hippocampus are minimally damaged and less involved in posttraumatic epileptogenesis. Such highly focal neocortical injury may at least partially explain why only mild spontaneous seizures are observed in the undercut model. On the other hand, the lack of widespread brain injury in this model can be advantageous in certain aspects: a more localized and consistent lesion may reduce variability and increase the possibility that epileptiform activity is actually originated from the cortical circuit of interest. The high consistency in producing hyperexcitable brain tissue not only is important for efficient study of epileptogenesis, but also may suggest modeling of common essential factors in the pathological process of epileptogenesis such as axonal sprouting, reorganization of cortical circuits, homeostatic synaptic plasticity, and altered GABAergic inhibition [22, 23, 26, 27, 44]. The key point is to take into account the advantages and limitations in choosing this unique model for addressing specific scientific questions.

In conclusion, we used continuous video/EEG monitoring to characterize epileptiform activities in the neocortical undercut model in mice. Spontaneous epileptic seizures were detected in 50% of mice in about 3–4 weeks after undercut lesion. The good percentage of PTE, relatively short latency from injury to seizure onset, and high consistency make this model useful and efficient for research on mechanism, prevention, and treatment of posttraumatic epilepsy.

## Supporting Information

S1 Fig An EEG trace showing a spontaneous seizure. This figure shows the EEG activity recorded simultaneously with S1 Video.
(TIF)

S1 Video An episode of behavioral seizure. This video shows an undercut mouse featuring sudden motion freeze that last for about 5–6 seconds and subsequent recovery.
(AVI)
